# The Potential of Cyclodextrins as Novel Active Pharmaceutical Ingredients: A Short Overview

**DOI:** 10.3390/molecules22010001

**Published:** 2016-12-25

**Authors:** Massimiliano Pio di Cagno

**Affiliations:** Drug Transport and Delivery Research Group, Department of Pharmacy, University of Tromsø, The Arctic University of Norway, 9019 Tromsø, Norway; massimiliano.p.cagno@uit.no; Tel.: +47-77-645-301

**Keywords:** cyclodextrins, inclusion complexes, solubilizing agents, cytotoxic activity, hypocholesterolemic agents, Niemann-Pick Type C disease treatment

## Abstract

Cyclodextrins (CDs) are cyclic oligosaccharides of natural origin that were discovered more than 100 years ago. The peculiar cone-like conformation of the sugar ring, expressing a lipophilic cavity and a hydrophilic external surface, allows these substances to spontaneously complex poorly soluble compounds in an aqueous environment. For more than 50 years, these substances have found applicability in the pharmaceutical and food industries as solubilizing agents for poorly soluble chemical entities. Nowadays, several research groups all over the world are investigating their potential as active pharmaceutical ingredients (APIs) for the treatment of several illnesses (e.g., hypercholesterolemia, cancer, Niemann-Pick Type C disease). The aim of this review is to briefly retrace cyclodextrins’ legacy as complexing agents and describe the current and future prospects of this class of chemical entities in pharmaceutics as new APIs.

## 1. Cyclodextrin Types and Characteristics

**Figure 1:**
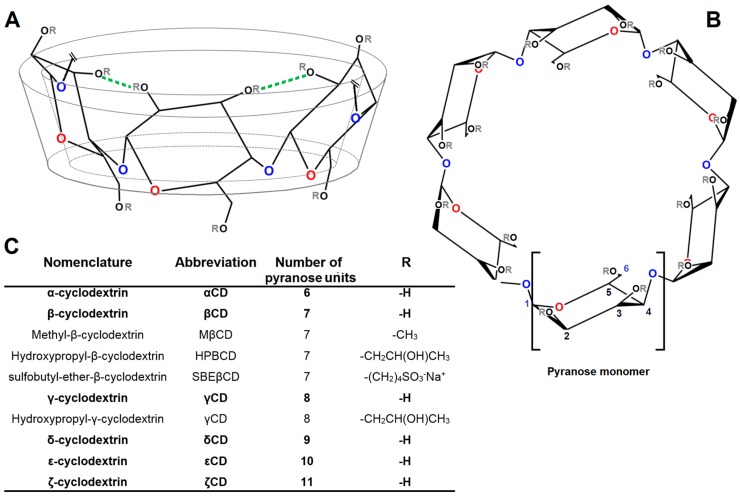
Front side prospect (**A**); top prospect (**B**); nomenclature and chemical characteristics (**C**) of most common cyclodextrins. The blue oxygens represent the etheric bonds 1–4 (inter-monomeric bonds), whereas, the red oxygens represents pyranose ether bonds 1–5 (intra-monomeric bonds). The green lines represent the intramolecular H-bonds forming in βCD when no substituent groups are present (R: -H).

Cyclodextrins (CDs) are chemical entities of natural origin, which are derived from bacterial degradation of starch through the metabolic action of cyclodextrin glycosyl transferase enzyme (GCTase). This peculiar enzyme is present in several species of alkalophilic bacillus species (e.g., Bacillus macerans [1]) and it is capable of catalyzing the synthesis of cyclic oligosaccharides, starting from amylose. The general structure of CDs is based on α-d-glucopyranose units linked 1 → 4, as in amylose. Three ring-types are common, where alpha-cyclodextrin (αCD) is composed of six, beta-cyclodextrin of seven (βCD), and gamma-cyclodextrin of eight glucose units (γCD). All CDs are shaped as trunked cones (Figure 1A), expressing a hydrophobic inner cavity and an external hydrophilic surface. Even though the chemical structure of CDs may appear rather simple, the complete characterization and understanding of CDs chemical properties and geometrical three-dimensional conformations was a research enterprise, which required more than half a century to be completed [2]. The first isolation of cyclodextrins was made in 1891 by Antoine Villiers, but it took more than 50 years to obtain the correct characterization of cyclohexaamylose (αCD) and cycloheptaamylose (βCD) molecular weighs [3]. The basic physicochemical characteristics of α-, β-, and γCDs—including a detailed description of cavity sizes and, for the first time, inclusion of complex formation tendency—was first published in 1954 by Friedrich Cramer [2,4]. Subsequently, Pulley and French [5] discovered bigger cyclodextrins composed of 9 (δCD), 10 (εCD), and 11 (ζCD) glucose units (Figure 1C). At present, cyclodextrins containing up to 31 glucose units have been purified and characterized and the existence of even larger cyclodextrins containing up to several hundreds of glycosyl units have been reported [6]. The most important physicochemical properties of α-, β-, and γCDs (most relevant CDs) are reported in Table 1.

As it can be seen, aqueous solubility of cyclic saccharides is much lower in comparison to similar acyclic sugar molecules. The reason of that is found in the high energy of crystal lattice owned by cyclic saccharides when in a solid state. Among the three most investigated CDs, βCD has the lowest solubility in water (approx. 0.016 M at 25 °C), followed by αCD (0.12 M) and γCD (0.17 M) [7]. It is quite interesting to see that βCD shows significantly less water solubility with respects to similar cyclic saccharides. This phenomenon is connected to intramolecular hydrogen bond formation between the hydroxyl groups of neighboring glucose units [8], as shown in Figure 1. This is reflected in the higher Gibbs free energy of dissolution owned by βCD in comparison to others (Table 1). Even though the hepta-pyranose ring results in poor aqueous solubility, the cavity diameter (6 to 6.5 Å) is of optimal size to accommodate a vast majority of chemical entities [9]. To increase the poor aqueous solubility of βCD, several derivatives have been synthetized substituting hydroxyl groups with other hydrophilic moieties such as sulfobutyl ether- (SBEβCD) and hydroxypropyl groups (HPβCD) (Figure 1C). The introduction of methyl groups (MβCD) also proved to be very efficient in increasing aqueous solubility of βCD due to the breakage of intramolecular hydrogen bonds [10]. Recently, polymeric derivatives of βCDs such as polyethylene glycol- (βCD-PEG) and dextran- βCD (βCD-dextran) have been developed to achieve higher aqueous solubility [11,12].

## 2. Inclusion Complex Formation and Its Investigation

**Figure 2:**
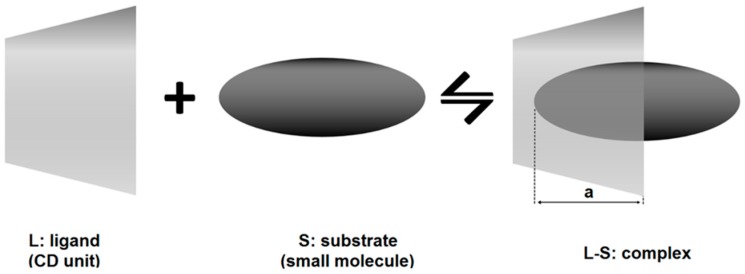
Schematic representation of the inclusion mechanism of complexation between CD (the ligand, L) and a small molecule (the substrate, S) in the case of 1:1 stoichiometry (molar ratio). The depth (a) that the substrate molecule can reach in the CD cavity varies according to the substrate physicochemical properties and CD steric hindrance.

When cyclodextrins (the ligand, L) are in aqueous solution together with compatible molecules (the substrate, S), they will spontaneously interact forming an inclusion complex (Figure 2).

If the stoichiometry of the complexation is 1:1 (Figure 2), the complexation equilibrium and its stability constant are expressed as:

(1) $$S+L\;\overset{K_{11}}{↔}SL$$

(2) $$K_{11}=\frac{\left[SL\right]}{\left[S\right]\left[L\right]}$$

where *SL* represents the complex formed between substrate and ligand and *K*_11_ the stability constant of the complex (generally expressed in M^−1^).

**Figure 3:**
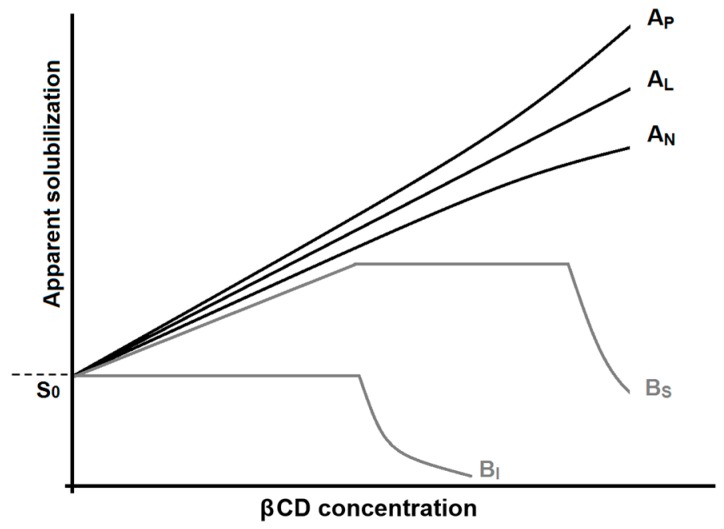
Phase-solubility diagrams associated with CDs. *S*_0_ represents the thermodynamic solubility of the chemical entity in the absence of CDs. If complexation is effective and the complex formed results water-soluble, the substrates’ apparent solubility increases (A types). If the complexation is inefficient or the complex formed is poorly soluble, the solubility of the substrate decreases (B type).

From the thermodynamic point of view, the Gibbs free energy (Δ*G*^0^) of complexation results are:

(3) $$\Delta G^{0}=-RTlnK_{11}=\Delta H^{0}-T\Delta S^{0}$$

where *R* is the gas constant, *T* is the absolute temperature, Δ*H*^0^ is the standard enthalpy of complexation, and Δ*S*^0^ is the standard entropy of complexation. The driving forces of spontaneous complexation are primarily the formation of non-ionic interaction (e.g., van der Waals interactions, hydrogen bonds formation) between the CD cavity and the binding site of the substrate (resulting in negative standard enthalpy) and the solvent effect (resulting in either positive or negative standard entropy) [13]. For the three most common CDs, *K*_11_ values vary from 0.1 M^−1^ for very weak interactions, up to 1,000,000 M^−1^ for very stable complexations. Moreover, α-, β-, and γCDs appear to be quite alike in their complexation abilities [14]. Unfortunately, standard βCD is of poor aqueous solubility (Table 1). Therefore, its direct usability is limited. Common techniques used to investigate the stability of the complex cyclodextrin-substrate are calorimetric analysis [15,16], nuclear magnetic resonance (NMR) [17,18], X-ray diffraction [19], and computational modelling [20]. An alternative classical method widely used to investigate the efficiency of CDs complexation with APIs is phase-solubility study [21,22]. This method is based on the paradigm that, if the CD is complexing a ligand in solution, this will result in solubilization of the API (i.e., higher apparent aqueous solubility of the substrate). Figure 3 shows all different types of phase solubility diagrams obtainable for CD complexation and solubilization of substrates.

When there is a linear dependence between the substrate (i.e., API molecule) concentration and the ligand (i.e., CD) concentration, the graphs are generally defined as A_L_ type [22]. In this case, the relation between concentration of CD and the API results in a linear regression. From this type of graph, knowing the thermodynamic solubility of the substrate (*S*_0_) and the slope of the linear regression, it is possible to estimate the equilibrium constant employing Equation (4) [13]:

(4) $$K_{1:1}=\frac{\left(slope\right)}{\;s_{0}\left(1-slope\right)}$$

**Figure 4:**
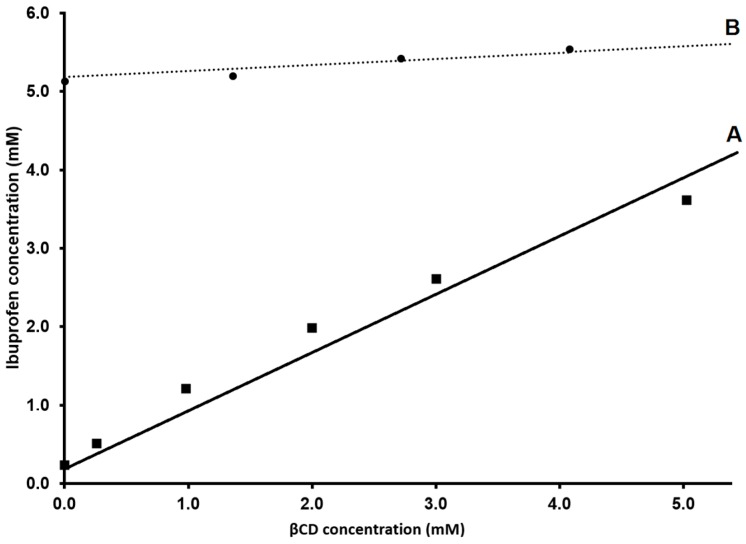
Phase-solubility profiles of IBU in the presence of βCD obtained at pH 3.8 (**A**) and at pH 7.4 (**B**). Personal communication data.

Phase-solubility study is a very efficient method for the evaluation of cyclodextrin solubilization of a substrate (i.e., increasing of apparent solubility of an API, Figure 3) but it shows limitations in stability constant determination [23]. Moreover, the choice of the condition of the aqueous media of complexation (e.g., pH, tonicity) is crucial for a correct estimation of *K*_1:1_ with this technique. For instance: in Figure 4, the phase-solubility diagram of ibuprofen (IBU, API commonly used as model drug for complexation studies [17,24,25]) in the presence of βCD is reported as an example. From Figure 4, it is clear that pH of the experiment has a huge impact on the phase-solubility profile of IBU and, consequently, on the equilibrium constant. In the case of IBU at pH 3.8 (Figure 4A), the calculated *K*_1:1_ results are 7.9 × 10^3^ M^−1^ (in good agreement with ITC data reported in the literature [17]). However, for the same complexation in neutral pH (Figure 4B), the equilibrium constant results calculated from the phase-solubility diagram are almost three orders of magnitude lower. Deviation from the linearity in a phase-solubility diagram indicates a deviation from a 1:1 stoichiometry of reaction. The deviation from linearity can be positive (A_P_) or negative (A_N_) (Figure 3). A positive or negative deviation form linearity implies that the molar ratio between ligand and substrate (CD and drug respectively) is not 1:1 [10].

**Figure 5:**
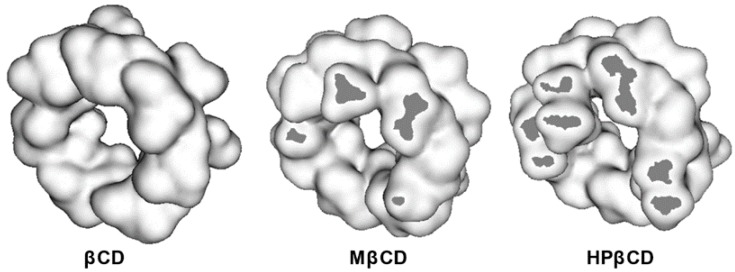
3D representation (figure adapted from [28]) of native βCD, MβCD, and HPβCD. Dark grey sections represent the steric hindrance generated by the different type of substituent group.

In this case, it is very difficult to predict from these diagrams the real stoichiometry of CD-drug complex formation. Positive deviation has been described by Loftsson et al. [26] to be primarily associated with formation of water-soluble aggregates through non-inclusion complexation with a stoichiometry drug/CD from 1:2 to 1:3. Negative deviation from linearity is in most cases very difficult to describe stoichiometrically. Quoting Nicol at al. [27], studies on non-linear drug solubility based on the many-body interaction theory “A_P_ behavior is due to the overriding drug-CD interaction, whereas A_N_ results from the overriding drug-induced weakening of CD-CD interactions”. If there is no increase or an increase followed by a drastic decrease of the substrate, solubility is measured with increasing concentration of CD, this generally indicates precipitation of the complex CD-drug. These diagrams are generally defined as B types (B_S_ and B_I_, Figure 3) [13]. It has been reported that complexation efficiency of CDs might, in some cases, be negatively influenced by the presence of substituent groups anchored to the cavity entrance [17]. This phenomenon is attributed to steric hindrance that reduces the depth that the substrate molecule can reach in the CD cavity (Figure 5, [28]).

## 3. Food and Drugs Relevance of CDs

**Figure 6:**
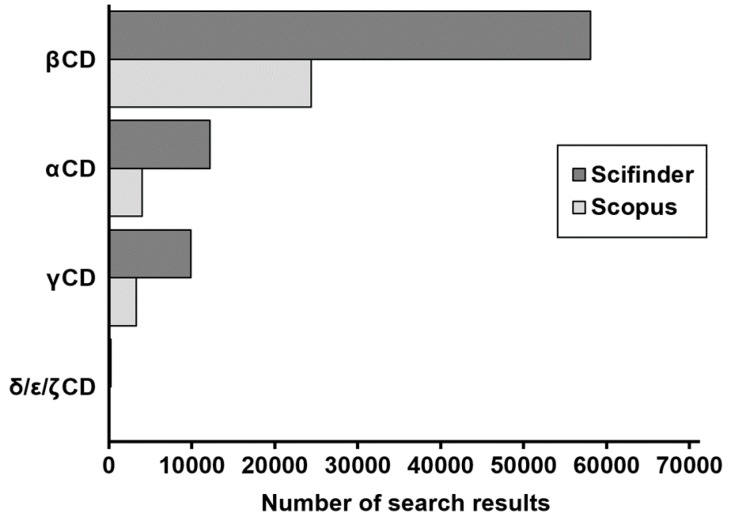
Number of search results obtained using as keyword the generic names of the most common CDs. Data collected from web search engines Scifinder (dark grey) and Scopus (light grey).

The interest of pharmaceutical research and food industry has been mostly pointed to hepta-pyranose ring types and, to a lower extent, α- and γCDs. Figure 6 reports the number of search results obtained by typing the generic names of the most common CDs as keywords. This diagram clearly shows that βCD (and its derivatives) are by far the most studied cyclic oligosaccharides, followed by αCD and γCD. Figure 6 also shows that larger cyclodextrins are currently very poorly studied.

The ability of CDs, and especially the strong ability of βCDs in forming stable inclusion complexes with poorly soluble and lipophilic moieties in aqueous environments, was immediately recognized to have big potentials in drug development and food industry. Cyclodextrins were first employed in the food industry in the 1970s, and from that time they have been mostly used as food additives for carrying food-related lipophiles (e.g., vitamins, aromas, and colorants) and for inhibiting light/heat mediated food degradation [29]. βCD was found suitable as a cholesterol-reducing agent in food of animal origin such as mink and egg [30,31]. The first pharmaceutical patent related to CDs and pharmaceutical applicability as complexing agents is dated 1953 [32]. Cyclodextrins are employed in pharmaceutical products primarily to increase water solubility of poorly soluble APIs, in order to enhance their bioavailabilities. Pharmaceutical products containing CDs comprise nasal spray, oral solutions, solid dosage forms, ocular and dermal formulations, suppositories, and parenteral solutions [33]. Currently, more than 40 pharmaceutical products containing cyclodextrin are available in the market worldwide, and the vast majority of them utilize βCD and its derivatives of higher water solubility such as HPβCD, MβCD, and SBEβCD [34]. Most of the βCD are approved from the European Medical Agency (EMA) for all administration pathways besides parenteral administration where only HPβCD and SBEβCD are approved [33]. MβCD application is limited to nasal and ocular administration due to side effects such as hemolysis. Even though MβCD has thermodynamically favorable complexation potentials for phospholipids [35], it is assumed that the hemolytic effect is primarily related to cholesterol segregation and depletion from plasma membranes of erythrocytes [36,37]. It is largely unknown, but CDs were first employed as therapeutic agents almost 30 years ago. In fact, in 1987 Carpenter and co-workers administered HPβCD to two siblings affected by an acute intoxication from retinoids (hypervitaminosis A). In this trial experiment, HPβCD proved to increase the urinary excretion of vitamin A, helping in the treatment of the acute symptoms of the intoxication [38].

The aim of the following sections is to present the reported interactions of βCDs with some biological substrates and the consequent in vivo and in vitro effects induced by these interactions. These effects (poorly highlighted until now) might lead to the development of βCDs as APIs.

## 4. Interaction of Cyclodextrins with Biologically Relevant Substrates

### 4.1. Interaction with Cholesterol and Lipids

The interaction of lipids and cholesterol with cyclodextrins has been extensively investigated in the last two decades. Loftsson and co-workers investigated the complexation reaction between cholesterol and different types of βCD in aqueous dispersion by phase-solubility diagrams [26]. It was shown that cholesterol had the highest affinity for the most lipophilic βCD (methylated one), but the lowest affinity for the very hydrophilic HPβCD and charged βCD (i.e., SBEβCD). From the phase-solubility diagrams of uncharged cyclodextrins, they estimated the formation of 1:2 drug/cyclodextrin complexes. These findings were later confirmed from Nishijo and co-workers [39]. In their study, they characterized the aqueous complex between cholesterol and different sugar rings of different cavity sizes (α-, β-, and γCD) using phase-solubility studies as well as NMR spectroscopy. Their results showed that only the methylated derivatives of βCD (MβCDs) were able to complex and solubilize cholesterol in an aqueous environment, whereas native βCD, αCD, and γCD cyclodextrin were not. More recent studies elucidated that native βCD is also capable of complexing and solubilizing cholesterol, but the effect is not measurable through phase-solubility study at room temperature [40] because of the precipitation of the poorly soluble βCD-cholesterol conjugates. This observation was confirmed by molecular dynamic simulations studies [41]. Highly hydrophilic polymeric derivative of standard native βCD (i.e., dextran derivative, βCD-dextran) showed comparable cholesterol-solubilization efficiency to MβCD [42]. For the same polymers, it was proven by isothermal titration calorimetry that the complexation efficiency of the βCD units was not negatively affected by the anchorage to the polymeric chain [43]. Recently, the ability of different types of βCD to deplete cholesterol (and cholesteryl esters) from phospholipid bilayers has been proven. Stelzl and co-workers [42] showed that cholesterol depletion from giant unilamellar vesicles (GUV) composed of phosphatidylcholine (PC) and cholesterol was a favorable process that spontaneously took place when the lipid bilayers were exposed to different types of βCD (primarily MβCD, βCD-dextran and, to a smaller extent, HPβCD) in a water environment. A similar study conducted by Litz and co-workers showed by fluorescence spectroscopy that cholesterol removal from GUVs was extremely fast (in the order of milliseconds), and it produced the formation of extended holes in the lipid bilayers (in vesicles composed by both saturated and unsaturated phospholipids) [44]. MβCD was found to have a strong and thermodynamically favorable inclusion affinity also for the phospholipids composing the vesicle bilayer (1-palmitoyl-2-oleoyl-sn-glycero-3-phosphocholine) [35]. Interestingly, Ao and co-workers [45] recently have shown that hydrophobic derivatives of βCDs (i.e., MβCD) are capable of disassembling low-density lipoprotein aggregates (LDL) by lipids (both phospholipids and cholesterol) segregation and they are significantly reducing the oxidation tendency of LDL (widely accepted as the key trigger of atherosclerosis).

### 4.2. Interaction with Proteins

Cyclodextrins have been found to be capable of interacting not only with lipophilic moieties but also with hydrophilic biological macromolecules such as proteins, effecting their stability and aggregation tendency [46]. Differential scanning calorimetry (DSC) of a range of proteins has shown that α- and βCDs can reduce the mean unfolding temperature of some biologically relevant proteins (e.g., lysozyme, ribonuclease A, ubiquitin, and yeast phosphoglycerate kinase). It has been suggested that this phenomenon is related to the inclusion of aromatic portions of some amino acids (e.g., tryptophan) from CDs, effecting protein stability [47]. The spontaneous inclusion tendency of βCDs of amino acids containing aromatic groups was later confirmed by a combined NMR and molecular docking approach [48]. Other studies showed that both MβCD and HPβCD were capable of forming spontaneous complexes with a portion of insulin, inducing a stabilizing/protecting effect in different aqueous environments and temperatures [49,50]. This stabilization effect also prevented aggregation of insulin molecules in solution. Another very interesting interaction that has been described is the interaction between βCDs and β-amyloid peptide [51]. Β-amyloid is the major constituent of the plaques forming in the human brain that induce Alzheimer’s disease (AD). It has been shown that βCDs can form inclusion complexes with portions of the peptide (where tyrosine and phenylalanine amino acids are located) [52]. This interaction sensibly reduces the aggregation tendency of this peptide in aqueous solution. 

All these spontaneous interactions between CDs and biologically relevant substrates induce evident effects when cyclodextrin is applied in vitro or administered in vivo.

## 5. Effect of Cyclodextrin In Vitro

Cholesterol is an essential component of plasma cell membranes. It is fundamental in keeping barrier integrity and its involvement in the growth and proliferation of cells has been described. In 1982, Irie and co-workers reported a lytic and shape-changing effect induced on human erythrocytes by some types of CD derivatives and in particular HPβCDs [53]. They observed a change in surface tension in the plasma membrane and they suggested that this phenomenon was due to segregation of important components of cell barriers (lipids and cholesterol in particular). In 1994, Learoy-Lechat and co-workers identified that, when eukaryotic cell lines (such as erythrocytes and murine leukemic cells P388) were incubated with significant amount of different α-, β-, and γCD (unmodified and hydroxypropylated), their viability was compromised [54]. The in vitro cytotoxicity of CDs was ranked as βCD > αCD > γCD (the same ranking was found for the hydroxypropylated derivatives). In accordance with Irie and co-workers, they also suggested that segregation of important components of cell membranes from CDs (in particular cholesterol) was the key factor in triggering hemolysis. More recently, an effect of CDs on plasma fibrinogen and a consequent effect on coagulation has also been described [55].

Another study performed by Kiss and co-workers [36] (focused primarily on both neutral and ionic derivatives of βCD) demonstrated a solid correlation between βCDs cytotoxicity and cholesterol segregation ability. In this regard, it has been shown that the depletion of cholesterol from cell membranes can lead to spontaneous activation of apoptotic/necrotic mechanisms [56,57,58,59]. However, it is still unclear which cellular mechanism promotes cell death after cholesterol depletion. One possible mechanism might be related to the perturbation of cell raft domains and the consequent alteration of the cascade of intramolecular signals regulated by them. In fact, the lipid raft domains of plasma membranes control several membrane receptors and regulate a number of intracellular signaling pathways [60]. Membrane lipid rafts have been implicated in the regulation of malignant cell proliferation, differentiation, apoptosis, and migration [61,62], suggesting that the alteration of these domains might lead to inhibition of metastatic cells growth/migration, also inducing cell death [63]. As mentioned earlier, hepta-sucrose rings showed ability in interaction with peptides and proteins. A recent study from Ren and co-workers [64] has shown that HPβCD owns strong inhibitory effect on the aggregation of amyloid-β peptides. They also have shown that administration of HPβCD on compromised neuroblastoma cells (i.e., previously exposed to β-amyloid peptide) significantly increases cell viability.

## 6. Potentials of Cyclodextrins in Therapy: In Vivo and in Human Evidence

### 6.1. Cyclodextrin Potentials on Atherosclerosis and Dyslipidemia Treatment

One of the predominant pharmacological effects that is reported in the literature for βCDs derivatives is their positive pharmacological activity on the cardiovascular system. In 1992. Irie and co-workers administered intravenous (IV) HPβCD to hyperlipidemic rabbit. They demonstrated a decrease in blood cholesterol levels after a single administration [53]. Sustained parenteral administration of HPβCD in animals increased cholesterol concentration in urines, inducing a positive effect on atherosclerotic plaques. In their 1995 study, Favier and co-workers [65] orally administered to male Wistar rats daily doses of βCD through animals’ regular food. Amazingly, they demonstrated that βCD acted as a very efficient hypocholesterolemic agent. They verified that the blood cholesterol content, as well as the concentrations of low density lipoprotein protein (LDL) and very low density lipoprotein (VLDL), were significantly reduced when βCD were administered through the food. Whereas concentration of high density lipoprotein (HDL) was less effected. A significant reduction in lipoprotein triglycerides content was also demonstrated, but predominantly when βCD were administered through a low-fat dietary regime.

Rivers and co-worker focused their attention on hypoxia and ischemia, demonstrating that the administration of HPβCD to rats after 30 min from the appearance of hypoxia/ischemia symptoms significantly reduced brain injuries [66]. The authors proposed HPβCD as a possible new treatment of cerebral ischemic injury. More recently, it has been shown that intraperitoneal administration of aqueous infusion of KLEPTOSE^®^ CRYSMEB (commercial name of MβCD from Rochètte Freres, Lestrem, France) were able to efficiently reduce atherosclerosis in ApoE^−/−^ male mice (class of mice often employed as in vivo models for atherosclerosis). Similar results were obtained by Zimmer and co-workers [67] on the same animal model but employing the more hydrophilic and FDA/EMA approved HPβCD. Both studies agreed that the mechanism by which βCD derivatives develop regression in atherosclerosis was a combination of alteration of lipid profile (i.e., hypocholesterolemic effect) and by influencing the inflammatory response that triggers atherosclerotic plaque formation (i.e., interaction with macrophages and lymphocytes). Zimmer and co-workers actually proposed HPβCD as a new treatment for atherosclerosis.

### 6.2. Cyclodextrins Potentials in Chemotherapy

As described previously, βCD and its derivatives of higher water solubility (i.e., MβCD and HPβCD) have shown interesting cytostatic/cytotoxic activity cholesterol-depletion mediated on different cell lines. However, just a handful of studies have been conducted to verify the potential of βCDs as APIs in chemotherapy. In 1998, Grosse and co-workers [68] demonstrated that intraperitoneal (IP) injections of aqueous solution of MβCD significantly inhibited the growth of human solid tumors implanted on mice (breast cancer and ovarian carcinoma cell lines). The antiproliferative effect of MβCD was comparable to the one measured for doxorubicin. Intratumoral (IT) injection of MβCD aqueous solutions resulted in an even higher antiproliferative effect [58]. More recently, Mohammed and co-workers proved that administration of tamoxifen in combination with MβCD significantly diminished (approx. 75% in normal diet fed animals) the size and weight of solid tumors in mice [69]. They also found out that, when animals were feed with a cholesterol rich dietary regime, no significant reduction in the size of the solid tumor was measurable when MβCD and/or tamoxifen where administered. This fact clearly indicates that cholesterol plays a fundamental role in tumor growth and that MβCD activity is almost certainly related to cholesterol depletion from the organism. Onodera and co-workers showed that folate-conjugated MβCD possessed an even higher antiproliferative activity on solid tumors (IT and IV injections) [70] as well as on melanoma (subcutaneous administration, SC) [58] with negligible side effects. The higher activity of these folate derivatives could be related to the higher number of folate receptors expressed by cancer cells in comparison to healthy cells (i.e., selectivity of action). Yokoo and coworkers showed that HPBCD also possessed antitumor action, demonstrating cell growth inhibition and apoptosis in leukemic cells in vitro and in vivo [71].

### 6.3. Cyclodextrin Potentials in the Treatment of Degenerative Brain Disease

CDs have been investigated as possible treatment for Niemann-Pick type C (NPC) disease. This syndrome is characterized by accumulation of esterified cholesterol and other lipids in cells, predominantly in liver and central nervous systems. This uncontrolled accumulation causes irreversible brain and liver damage that ultimately leads to death. Currently, no efficient medicine is available for the treatment of NPC. IV administration of HPβCD to symptomatic Npc1^−/−^ mice (standard animal model for NPC) proved to sensibly delay the onset of neurological symptoms in the animals [72]. Later study showed that SC and IP administration of HPβCD to NPC diseased mice could reduce neuronal cholesterol accumulation [73]. Interestingly, the life span of animals was prolonged to the same extent as for miglustat administration (the only API approved for NPC treatment). Detailed histological investigations also revealed a clear reduction in cholesterol accumulation in the most sensitive organs of Npc1^−/−^ mice (liver, spleen, and kidneys) after weekly administration of HPβCD (SC injections) [74]. A similar study conducted by Tanaka et al. identified a significant increase in life span of Npc1^−/−^ of mice after daily SC administration of HPβCD [75]. In recent years, several investigative new drug application documents for cyclodextrin treatment for Niemann Pick Type C disease were filed [76] (and approved by FDA). Intravenous administration of HPβCD in human patients proved to be not so effective due to the moderate permeability of the pyranose derivatives through the blood brain barrier (BBB) [77]. In this regard, intrathecal infusion of βCDs resulted in a much more efficient administration pathway and, nowadays, several pharmaceutical trials employing HPβCD for NPC on humans are currently underway in several countries [78]. 

Another extremely severe neurological disorder that is not treatable by current medicines is Alzheimer Disease (AD). Yao and co-workers [79] proved that weekly subcutaneous injection of HPβCD in mice model for AD (TG19959) could significantly improve memory deficits and reduce β-amyloid peptide neuronal deposition. It was suggested that the reduction in β-amyloid aggregation/deposition in the neurons could be related to alteration in cholesterol metabolism. Subsequent studies have suggested that HPβCD probably interacts with β-amyloid peptide, reducing its aggregation and deposition tendency [64]. Even though the mechanism of action is not completely understood yet, HPβCD shows potential for the treatment of AD.

A summary of the cyclodextrins that have been investigated as possible active pharmaceutical ingredients in vivo (and in humans) is reported in Table 2. 

## 7. Conclusions

In the last three decades, cyclodextrin derivatives have shown tremendous potential for the treatment for different diseases where available medicines in the market are failing to provide proper treatment. Despite the promising results achieved in the last years, the interest of the pharmaceutical community and industries in developing CDs as active pharmaceutical ingredients remain regrettably low. As for the treatment of NPC, it is desirable that, cyclodextrins that are already approved from American and European medical agencies could be promptly used in human trials to verify their real potential as therapeutic agents. Moreover, the development of new CD types (e.g., polymeric cyclodextrins) is to be encouraged, because it might lead to the discovery of the ultimate cure for diseases such as atherosclerosis, cancer, and degenerative brain diseases that remain, at present, still of poor treatability and considerable lethality.

## Figures and Tables

**Table 1:** Some physicochemical properties of the most relevant CDs (α-, β-, and γCD) at 25 °C.

	αCD	βCD	γCD
Cavity diameter (Å)	≈5.2	≈6.6	≈8.4
Cavity volume (Å^3^)	100	160	250
Water molecules in the cavity (n^r^)	2.5	5.0	8.5
Aqueous solubility (M)	0.12	0.016	0.17
Gibbs free energy of dissolution (kJ/mol)	15	20	14

**Table 2:** Types of cyclodextrins that have been investigated as possible active pharmaceutical ingredients in vivo (and in humans *).

Cyclodextrin Type	Hypervitaminosis A	Dyslipidemia and Atherosclerosis	Chemotherapy	NPC	AD
βCD		X			
MβCD		X	X		
HPβCD	X *	X	X	X *	X

## Abbreviations

The following abbreviations are used in this manuscript:

API	active pharmaceutical ingredient
CD	cyclodextrin
αCD	alpha-cyclodextrin
βCD	beta-cyclodextrin
HPβCD	hydroxypropyl-beta-cyclodextrin
MβCD	methyl-beta-cyclodextrin
SBEβCD	sulfobutyl-ether-beta-cyclodextrin
γCD	gamma-cyclodextrin
IP	intra peritoneal
IV	intra venous
SC	subcutaneous
NPC	Niemann–Pick type C disease
AD	Alzheimer’s disease
